# FGF12 Enhances Prostate Cancer Cell Survival via the YB1-lncRNA Axis

**DOI:** 10.3390/cells14221828

**Published:** 2025-11-20

**Authors:** Zechao Huang, Sonia H. Y. Kung, Hans Adomat, Htoo Zarni Oo, Connor Forbes, Faraz Hach, Xuesen Dong

**Affiliations:** 1 The Vancouver Prostate Centre, Vancouver General Hospital, 2660 Oak Street, Vancouver, BC V6H 3Z6, Canada; 2 Department of Urologic Sciences, Faculty of Medicine, University of British Columbia, 2775 Laurel Street, Vancouver, BC V5Z 1M9, Canada

**Keywords:** treatment-induced neuroendocrine prostate cancer (t-NEPC), fibroblast growth factor 12 (FGF12), post-transcriptional regulation, Y-box binding protein 1 (YB1), long noncoding RNAs (lncRNAs)

## Abstract

**Highlights:**

**What are the main findings?**

- FGF12 is upregulated in t-NEPC across different models.
- FGF12 enhances PCa cell survival under chemotherapeutic stress by interacting with YB1 and promoting YB1-mediated binding and stabilization of oncogenic lncRNAs, NEAT1 and MALAT1.

**What is the implication of the main finding?**

- These findings reveal a novel FGF12-YB1-lncRNA axis in advanced PCa.
- Targeting this signaling axis could provide new therapeutic opportunities.

**Abstract:**

Treatment-induced neuroendocrine prostate cancer (t-NEPC) is a highly aggressive and therapy-resistant subtype of prostate cancer characterized by lineage plasticity and poor response to standard chemotherapy and androgen deprivation therapy. Although transcriptional mechanisms driving t-NEPC have been extensively studied, the contribution of post-transcriptional regulation remains less defined. Here, we report fibroblast growth factor 12 (FGF12) as a critical post-transcriptional regulator of t-NEPC progression. Transcriptomic analyses of patient biopsies, patient-derived xenografts, and prostate cancer cell models consistently demonstrated elevated FGF12 expression in t-NEPC, which was further validated by immunohistochemistry in archival specimens. Functional assays revealed that FGF12 expression conferred survival of cancer cells to chemotherapeutic agents, including etoposide and camptothecin. Integrative RNA sequencing and affinity purification–mass spectrometry showed that FGF12 mediates these functions mainly through interaction with the RNA-binding protein YB1, leading to stabilization of oncogenic long noncoding RNAs, including NEAT1 and MALAT1, whereas RNA silencing of YB1 abrogated the ability of FGF12 to upregulate these transcripts. Collectively, these findings uncover a previously unrecognized FGF12-YB1-lncRNA signaling axis that drives t-NEPC progression. Targeting this pathway may provide new therapeutic opportunities for patients with this aggressive disease.

## 1. Introduction

Castration-resistant prostate cancer (CRPC) is a lethal form of prostate cancer (PCa) that emerges following the failure of androgen deprivation therapy (ADT), the mainstay treatment for advanced disease [1]. Driven by therapeutic pressure from ADT, CRPC develop into various clinically and molecularly heterogeneous conditions, including amphicrine, AR-low, double-negative, neuroendocrine, and small cell phenotypes [2]. Among these, treatment-induced neuroendocrine prostate cancer (t-NEPC) represents a highly aggressive and treatment-resistant subtype [3]. Clinically, t-NEPC is characterized by rapid disease progression, low prostate-specific antigen (PSA) levels, and resistance to androgen deprivation therapies [3]. The progression of t-NEPC can be regulated via alterations in the genome, epigenome, and transcription [4,5,6,7], underscoring the complexity of t-NEPC progression. However, the role of post-transcriptional regulators and RNA-binding proteins during this process still remains unclear.

Fibroblast growth factor 12 (FGF12) is a member of the fibroblast growth factor (FGF) family, whose members are broadly involved in embryonic development, tissue repair, and tumor growth [8]. Unlike other classic FGFs, FGF12 belongs to the fibroblast growth factor homologous factor (FHF) subfamily, in which proteins mainly act intracellularly [9,10]. The most well-documented function of FGF12 is the modulation of sodium channels [11], but additional studies suggest broader cellular roles [8]. FGF12 was reported to negatively regulate NF-κB functions by interacting with NF-κB essential modulator (NEMO) [12]. FGF12 was also reported to be a novel component in nucleolar ribosome biogenesis machinery, where it is required for the interaction between NOLC1 and TCOF1 for ribosome assembly [13]. Furthermore, a recent study proved that FGF12 can be secreted into the extracellular space to bind to FGF receptors and activate anti-apoptotic responses of the cells [14]. These molecular function analyses supported the linkage of FGF12 with tumor progression. Elevated FGF12 expression has been observed in gastric cancer and esophageal squamous cell carcinoma (ESCC) [15,16]. High FGF12 expression in early-stage non-small cell lung cancers was associated with poorer overall survival [17]. In endometrial stromal sarcoma (ESS), high FGF12 expression is observed in a cellular subpopulation associated with poor prognosis [18]. These observations raise the possibility that FGF12 may have oncogenic roles across multiple tumor types. However, whether FGF12 contributes to therapy resistance or lineage plasticity of PCa, particularly in t-NEPC, has not been investigated.

In this study, we reported an upregulation of FGF12 in several CRPC tumor biopsies and t-NEPC tumor cells. FGF12 does not enhance PCa cell proliferation or induce NE differentiation, but enables PCa cells to survive under chemotherapeutic agents, including etoposide and camptothecin. We found that this FGF12 action is mainly mediated through the RNA-binding protein YB1, which enhances the stabilization of several oncogenic long noncoding RNAs (lncRNAs), including NEAT1 and MALAT1. Importantly, RNA silencing of YB1 abolished the ability of FGF12 to upregulate these transcripts, confirming a functional dependence on YB1. Together, these studies uncover a previously undefined FGF12-YB1-lncRNA signaling axis as a potential driver of t-NEPC progression.

## 2. Materials and Methods

### 2.1. Cell Culture and Reagents

LNCaP, Du145, and PC3 PCa cell lines were purchased from ATCC (Manassas, VA, USA). They were cultured in RPMI-1640 medium (Gibco, Waltham, MA, USA) or DMEM medium supplemented with 10% fetal bovine serum (FBS; Gibco, Waltham, MA, USA) under 5% CO_2_ at 37 °C. MR49F PCa cell line was provided by Dr. Martin Gleave (Vancouver Prostate Centre, Vancouver, BC, Canada) and was maintained in RPMI-1640 medium with 10% FBS and 10 μM enzalutamide [19]. The LN95 PCa cell line was a generous gift from Dr. Alan Meeker (Johns Hopkins University, Baltimore, MD, USA) [20], and the LNNE PCa cell line was generated as previously reported [21]. Both LN95 and LNNE cells were cultured in phenol-red-free RPMI-1640 medium (Gibco, Waltham, MA, USA) supplemented with 5% charcoal-stripped serum (CSS) (Hyclone, Logan, UT, USA) under 5% CO_2_ at 37 °C. All cell lines were tested and confirmed to be mycoplasma-free.

### 2.2. Public RNA-Seq Data Analysis

Unsupervised clustering analysis on RNA-seq data of five cell lines (LNCaP + DHT, LNCaP − DHT, LN95, MR49F, LNNE) was performed by using SRplot [22]. Public RNA-seq datasets were obtained from GEO and other published sources, including patients, patient-derived xenografts (PDX), gene-engineered mouse model (GEMM), and cell models. Expression levels of FGF12 were analyzed using normalized FPKM/Feature count values.

### 2.3. Quantitative Real-Time PCR (qRT-PCR)

Total RNA was extracted using TRIzol reagent (Invitrogen, Carlsbad, CA, USA) following the manufacturer’s instructions. RNA concentration and purity were measured by a NanoDrop spectrophotometer (Thermo Fisher Scientific, Waltham, MA, USA). For cDNA synthesis, 2 µg of total RNA was reverse-transcribed using random hexamers and SuperScript II (Invitrogen, Carlsbad, CA, USA). Quantitative PCR was performed using universal SYBR Green Mix (ABclonal, Woburn, MA, USA) on a QuantStudio 7 Pro Real-Time PCR System (Applied Biosystems, Foster City, CA, USA). Each reaction was run in triplicate. Gene expression was normalized to GAPDH, and fold changes were calculated using the 2^−ΔΔCt^ method. Primer sequences used for qRT-PCR are listed in Supplementary Table S1.

### 2.4. Western Blotting

Cells were lysed in a buffer containing 50 mM Tris, pH 8.0, 150 mM NaCl, 1% NP-40, 0.5% sodium deoxycholate, 1% SDS, and proteinase & phosphatase inhibitors (Roche, Indianapolis, IN, USA). Equal amounts of protein (50 µg) were separated by SDS-PAGE and transferred to PVDF membranes (Millipore Sigma, Jaffrey, NH, USA). Membranes were blocked with Intercept (PBS) Blocking Buffer (LI-COR, Lincoln, NE, USA) for one hour and incubated with specific primary antibodies against FGF12 (1:1000 dilution), YB1 (1:1000 dilution), and β-actin (1:1000 dilution) overnight (see Supplementary Table S2), followed by washing and one-hour specific secondary antibody treatment (1:10,000 dilution) (see Supplementary Table S2).

### 2.5. Immunohistochemistry (IHC)

IHC staining of FGF12 on a PCa tissue microarray (TMA) was performed at the Molecular Pathology Core of Vancouver Prostate Centre. The TMA contains 160 tissue cores from 80 patients, including untreated prostate adenocarcinoma (AdPC) (*n* = 50), NHT-treated AdPC (*n* = 30), CRPC (*n* = 58), and NEPC tumors (*n* = 22). IHC was performed by using the Ventana DISCOVERY ULTRA autostainer. The TMA section underwent antigen retrieval in CC1 (Ventana), a tris-based buffer, for 32 min at 95 °C and was then incubated with the FGF12 antibody (Proteintech 13784-1-AP, 1:250 dilution) for 32 min at room temperature. Detection was performed with DISCOVERY Anti-Rabbit HQ, DISCOVERY Anti-HQ HRP, and DISCOVERY ChromoMap DAB Kit (Ventana). The Leica Aperio AT2 scanning system (Leica Microsystems, Vista, CA, USA) was used to digitize the slides at a magnification of 40×. The images were subsequently stored in eSlide Manager (Leica Microsystems, Vista, CA, USA) at the Vancouver Prostate Centre. Pathological evaluation of these tumors was performed independently by 3 observers.

### 2.6. Plasmid Construction and Cell Transfection

Human FGF12-cloned pcDNA3.1(+) vector and empty pcDNA3.1(+) vector were purchased from GenScript (Piscataway, NJ, USA). LNCaP cells were transiently transfected with FGF12 or empty vector using Lipofectamine 3000 (Invitrogen) following the manufacturer’s protocol to generate FGF12-expressing (FGF12) and control cell lines. FGF12 expression was confirmed by qRT-PCR and Western blotting 48 h post-transfection.

### 2.7. Drug Treatment and Cell Viability Assays

LNCaP cells were treated with etoposide (0–5 μM) or camptothecin (0–50 nM) for 72 h on 96-well plates. Cell proliferation rates were measured by using Incucyte^®^ Live-Cell Analysis System (Sartorius, Ann Arbor, MI, USA) following the manufacturer’s protocol. Data of cell viability were normalized to 0 h and 0 concentration. IC50 values were calculated using nonlinear regression in GraphPad Prism 9.0.0.

### 2.8. RNA-Seq and Gene Set Enrichment Analysis (GSEA)

Total RNA from LNCaP cells was extracted using PureLink^®^ RNA Mini Kit (Invitrogen, Carlsbad, CA, USA) and submitted to Innomics Inc. (Sunnyvale, CA, USA) for total RNA-seq. Differential gene expression was analyzed with fold change and *p*-value. GSEA was conducted using the GSEA software 4.3.3 (Broad Institute).

### 2.9. Co-Immunoprecipitation (Co-IP)

Protein lysates from LNCaP FGF12 and control cells were incubated with normal IgG (Santa Cruz Biotechnology, Dallas, TX, USA) and anti-Flag antibody (see Supplementary Table S2), followed by pull-down using Protein A/G agarose beads (Santa Cruz Biotechnology, Dallas, TX, USA). Eluted proteins were subjected to SDS-PAGE and Coomassie staining. The proteins eluted were also analyzed by mass spectrometry at the Vancouver Prostate Centre. Data of unique peptides were used for analysis. YB1 was identified and validated by Western blotting.

### 2.10. RNA Co-Immunoprecipitation (RIP)

Lysates from LNCaP FGF12 and control cells were incubated with normal IgG (Santa Cruz Biotechnology, Dallas, TX, USA) and anti-YB1 antibodies, followed by pull-down using Protein A/G agarose beads (Santa Cruz Biotechnology, Dallas, TX, USA). 10% of the input was saved before pull-down. RNA from input and RNA after pull-down were extracted using PureLink^®^ RNA Mini Kit (Invitrogen, Carlsbad, CA, USA). RNA extracted was analyzed by qRT-PCR for NEAT1 and MALAT1 and calculated with the %Input method (%Input = 100 × 2^(Ct input−Ct IP)^ × 10 (percentage of input)). IP of YB1 was validated by Western blotting.

### 2.11. siRNA Knockdown

YB1 siRNA (#1 (predesigned): SASI_HS01_00017065, #2 (custom sequence): rUUCGUUGCGAUGACCUUCU[dT][dT]; rAGAAGGUCAUCGCAACGAA[dT][dT]) were purchased from MilliporeSigma (The Woodlands, TX, USA). Silencer^®^ Select Negative Control #1 siRNA (Invitrogen, Carlsbad, CA, USA) was used as a negative control. LNCaP cells (FGF12 and control) were transfected with YB1 siRNA (#1+#2) or negative control siRNA using Lipofectamine RNAiMAX (Invitrogen, Carlsbad, CA, USA) following the manufacturer’s protocol. RNA was extracted and analyzed by qRT-PCR to assess the expression of YBX1, NEAT1, and MALAT1. Knockdown efficiency of YB1 was validated by Western blotting.

### 2.12. Statistics

All results are expressed as the mean ± standard deviation (SD). Statistical analyses and data visualization were done using GraphPad Prism 9.0.0 and SRplot [22]. Student *t*-tests or one-way ANOVA were utilized to determine differences between groups. *p*-value < 0.05 is considered significant.

## 3. Results

### 3.1. FGF12 Is Upregulated in Different NEPC Models

**Figure 1:**
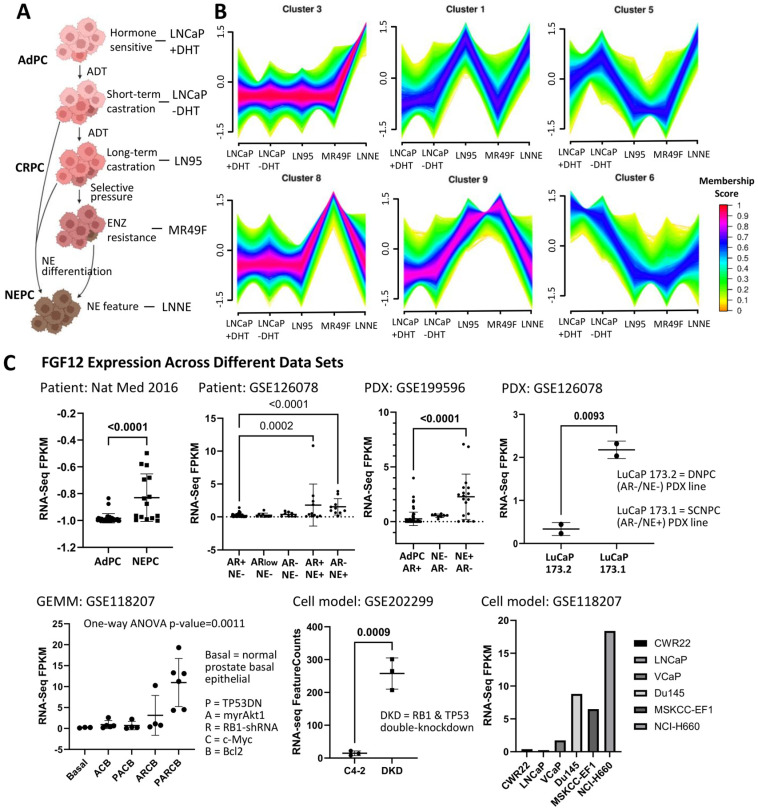
FGF12 is upregulated in t-NEPC. (**A**) Schematic showing progression of PCa from AdPC to NEPC. (**B**) Gene expression trend analysis from RNA-seq data of five PCa cell lines: LNCaP + DHT, LNCaP − DHT, LN95, MR49F, and LNNE. The plot shows expression patterns across gene clusters. FGF12 is located in Cluster 3, characterized by genes with marked upregulation in the NEPC cell line, LNNE, compared to the other cell lines. Data are presented as log2 FPKM values normalized by gene-wise Z-score. (**C**) RNA-seq analyses comparing FGF12 RNA expression levels among AdPC and NEPC with patient samples (Nat Med 2016 and GSE126078), PDXs (GSE199596 and GSE126078), GEMM (GSE118207), and cell models (GSE200299 and GSE118207). Expression levels are shown as log2 FPKM in dot plots, grouped by disease types. Statistical significance was assessed by *t*-test; *p* < 0.05 was considered significant.

To explore the potential genes involved in PCa progression, we compared transcriptomic changes across a series of well-established PCa cell line models representing different stages of PCa progression. As illustrated in Figure 1A, the progression model includes hormone-sensitive PCa (LNCaP + DHT), short-term androgen deprivation (LNCaP − DHT), long-term androgen deprivation (LN95), enzalutamide-resistant CRPC (MR49F), and t-NEPC (LNNE). We performed unsupervised clustering analysis on RNA-seq data from these five cell lines to identify gene expression patterns associated with disease progression. A total of nine distinct expression clusters were identified (see Supplementary Table S3 and Supplementary Figure S1), among which Cluster 3 exhibited a unique pattern characterized by low expression in LNCaP + DHT, − DHT, LN95, and MR49F, but markedly higher expression in LNNE (Figure 1B). This suggests that genes in Cluster 3 may be associated with the NE phenotype. Particularly, FGF12 was included in this cluster, implicating its role as a potential t-NEPC-related gene.

To validate the association of FGF12 with t-NEPC, we analyzed its expression across multiple independent RNA-seq datasets in different t-NEPC models (Figure 1C). We first examined RNA-seq data from patient biopsies, where a few cohorts are currently available. In a multi-institutional patient cohort [23], FGF12 expression was significantly elevated in t-NEPC tumors compared to AdPC, suggesting its clinical relevance in advanced disease. Consistently, in another patient cohort analyzing 98 patients (GSE126078) [24], FGF12 expression also exhibited upregulation in NE-positive cases. To further support these findings, we extended the analysis to PDX models [24,25]. In GSE199596 [25], FGF12 expression was markedly higher in NE-positive, AR-negative PDX lines. In GSE126078 [24], which included PDXs derived from the same parental tumor (LuCaP 173), the small-cell neuroendocrine PCa (SCNPC) line (LuCaP 173.1) exhibited higher FGF12 expression than the double-negative PCa (DNPC) line (LuCaP 173.2), supporting its upregulation in t-NEPC in vivo. Turning into GEMM of t-NEPC (GSE118207) [26], FGF12 expression was increased in tumors driven by the PARCB combination (dominant-negative TP53, myrAKT1, RB1 loss, c-MYC, and BCL2), indicating its association with t-NEPC phenotypes. Furthermore, in cell line models, analysis of GSE202299 [27] showed upregulation of FGF12 in C4-2 cells with dual knockdown (DKD) of TP53 and RB1. Also, in dataset GSE118207 [26], FGF12 expression was elevated in NEPC cell lines, such as NCI-H660, compared to AdPC cell lines. These results highlight FGF12 as a gene consistently upregulated in t-NEPC across cell line, mouse, PDX, and patient models, suggesting a potential role of FGF12 as a biomarker or functional driver in advanced PCa.

### 3.2. FGF12 Expression Is Elevated in NEPC Cell Line and Clinical Samples

**Figure 2:**
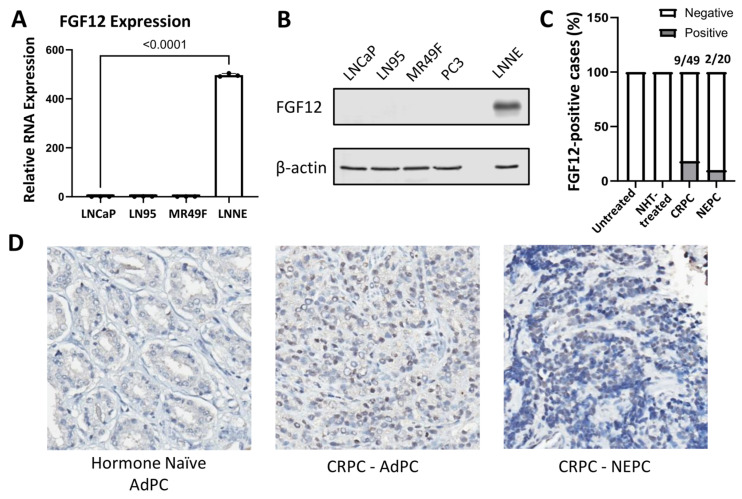
Experimental validation of FGF12 elevation in NEPC cell line and clinical samples. (**A**) qRT-PCR analysis of FGF12 mRNA levels in PCa cell lines (LNCaP, LN95, MR49F, and LNNE). Data normalized to GAPDH and presented as mean ± SD from three independent experiments. Statistical significance was determined by one-way ANOVA; *p* < 0.05 was considered significant. (**B**) Western blotting analysis of FGF12 protein expression in PCa cell lines (LNCaP, LN95, MR49F, PC3, and LNNE); β-actin serves as a loading control. (**C**) Immunohistochemical staining result of FGF12 in PCa patient tissues. (**D**) Representative images of Hormone Naïve AdPC, CRPC-AdPC, and NEPC cases.

We next performed experimental validation of FGF12 expression at both mRNA and protein levels. qRT-PCR analysis showed that FGF12 transcript levels were markedly upregulated in t-NEPC LNNE cells compared with AdPC and CRPC cell lines (LNCaP, LN95, and MR49F) (Figure 2A). Consistently, Western blotting analysis confirmed higher FGF12 protein expression in LNNE cells, whereas AdPC and CRPC cell lines, including LNCaP, LN95, MR49F, and PC3, exhibited no detectable FGF12 (Figure 2B). To extend these findings to clinical specimens, immunohistochemistry (IHC) was performed on a PCa tissue cohort. No FGF12 staining was detected in untreated (0/45) or NHT-treated (0/22) AdPC samples. In contrast, positive cytoplasmic staining was observed in 9 of 49 CRPC-AdPC cases (18%) and in 2 of 20 NEPC cases (10%) (Figure 2C). Selected representative images displayed positive staining signals in the CRPC-NEPC sample, whereas hormone-naïve AdPC tissue lacked staining signals (Figure 2D). Together, these results demonstrate that FGF12 expression is specifically elevated in a subset of CRPC and NEPC tumors, consistent with its higher expression in in vitro t-NEPC cell models.

### 3.3. FGF12 Expression Enhances LNCaP Cell Survival Under Stress

**Figure 3:**
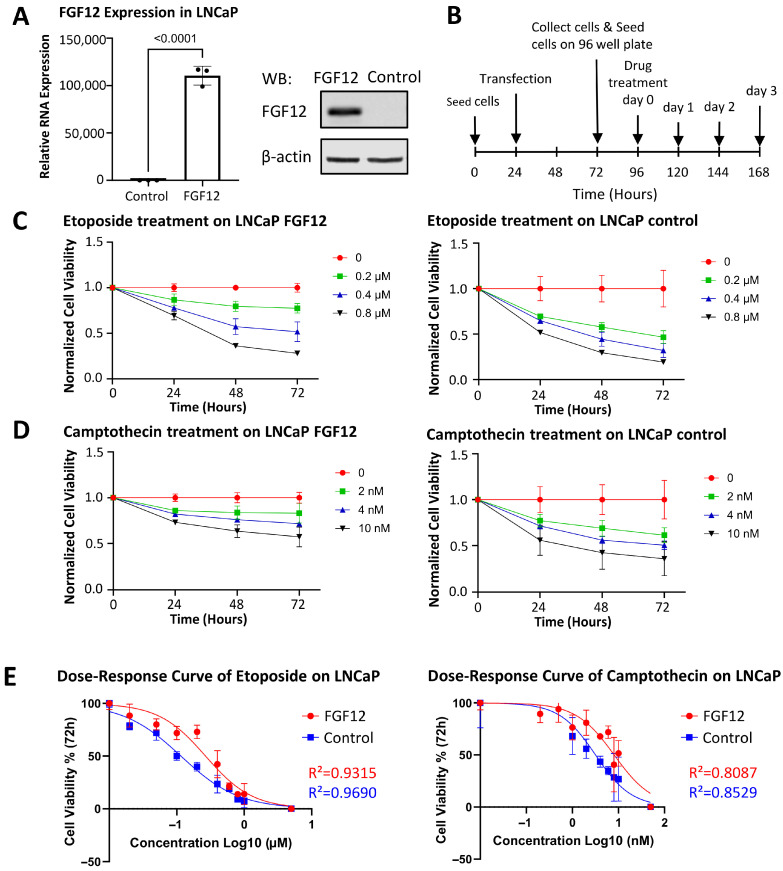
FGF12 enhances LNCaP cell survival under chemotherapeutics. (**A**) Validation of FGF12 expression in LNCaP cells. qRT-PCR (left) shows significant upregulation of FGF12 mRNA in FGF12 cells compared to control cells. Western blotting (right) confirms increased FGF12 protein levels; β-actin serves as a loading control. (**B**) Schematic timeline of transfection and drug treatment process. (**C**) Time-dependent responses of etoposide in LNCaP cells (FGF12 and control). Data of cell viability are normalized to 0 h and 0 concentration. (**D**) Time-dependent responses of camptothecin in LNCaP cells (FGF12 and control). Data of cell viability are normalized to 0 h and 0 concentration. (**E**) Dose–response curves of chemotherapeutic drugs (etoposide/camptothecin) in LNCaP cells (FGF12 and control) at 72 h. Data of cell viability are normalized to 0 h and 0 concentration. Non-linear regression is performed by GraphPad Prism to fit the dose–response curve. All the data in the figure are presented as mean ± SD from three independent experiments (*n* = 3).

Since upregulation of FGF12 is associated with CRPC/t-NEPC progression, we applied gain-of-function approaches by transiently expressing FGF12 in LNCaP cells to investigate its functional roles. Successful expression of FGF12 was confirmed by qRT-PCR and Western blotting analysis, which demonstrated a significant increase in FGF12 mRNA and protein levels in FGF12 cells compared to control cells (Figure 3A). Given its upregulation in t-NEPC and potential involvement in aggressive tumor phenotypes, we considered three possible outcomes of FGF12 expression: (1) induction of NE differentiation; (2) promotion of cancer cell proliferation; (3) reduced sensitivity to chemotherapeutic reagent treatments.

We first assessed whether FGF12 induces NE differentiation. qRT-PCR analysis showed that FGF12 did not change the expression of NE markers (CHGA and SYP) and luminal markers (AR, FOXA1, and CK8) (Supplementary Figure S2), suggesting that FGF12 does not directly regulate NE differentiation of AdPC cells. Next, we examined the effect of FGF12 on cancer cell growth. Gain-of-function of FGF12 in LNCaP cells modestly reduced the cell proliferation rate (Supplementary Figure S3), indicating that FGF12 does not promote PCa cell growth. Consistently, Western blotting analysis showed no detectable change in ERK phosphorylation by FGF12 (Supplementary Figure S4), suggesting that FGF12 does not activate canonical MAPK signaling pathways associated with proliferation.

Finally, we evaluated the impact of FGF12 on chemotherapeutic response. Figure 3B shows the schematic of the transfection and drug treatment workflow. Time-dependent cell viability assays revealed that LNCaP cells expressing FGF12 maintained higher viability than the control cells upon exposure to the chemotherapy drugs etoposide and camptothecin (Figure 3C,D). At various etoposide concentrations, LNCaP cells expressing FGF12 (Figure 3C, left) showed higher viability than control cells (Figure 3C, right) after 72 h of treatment. Similarly, under different camptothecin concentrations, LNCaP cells expressing FGF12 (Figure 3D, left) exhibited higher viability than control LNCaP cells (Figure 3D, right). Dose–response analyses after 72 h of treatment showed a rightward shift of the curves for FGF12-expressing cells relative to controls under both etoposide and camptothecin conditions (Figure 3E). Nonlinear regression analysis indicated that FGF12 increased IC50 values from 0.1155 μM to 0.2675 μM for etoposide and from 3.180 nM to 8.069 nM for camptothecin in LNCaP cells, confirming that FGF12 enhanced PCa cells to tolerate these drug treatments. Taken together, these results indicate that while FGF12 does not promote NE differentiation or accelerate proliferation, it significantly enhances survival under stress conditions in PCa cells.

### 3.4. Transcriptomic Profiling Reveals FGF12 Regulates lncRNAs and YB1-Related Gene Set

**Figure 4:**
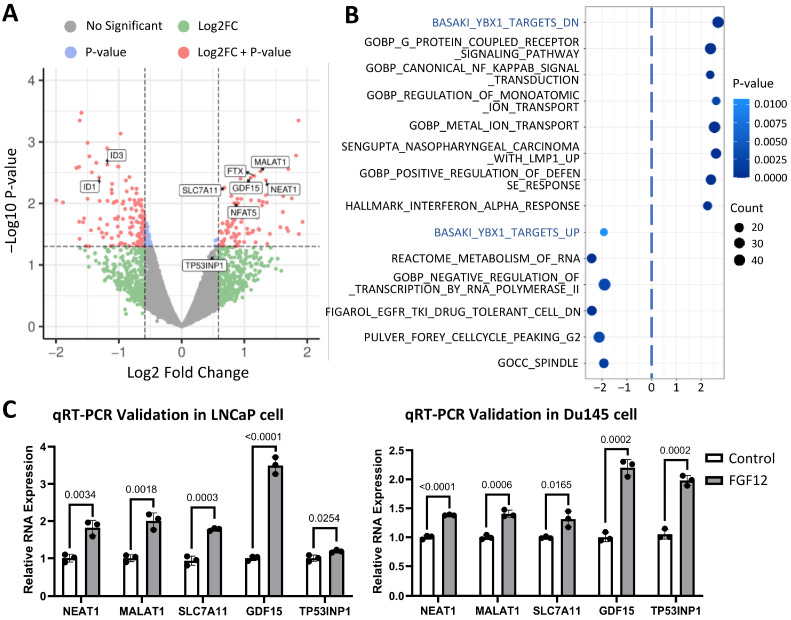
RNA-seq analysis of FGF12-expressing LNCaP cells reveals upregulation of lncRNA and activation of YB1-related gene set. (**A**) Volcano plot shows genes significantly changed in FGF12-expressing cells compared to control cells. Fold change cutoff at ±1.5, *p*-value cutoff at 0.05. (**B**) Gene Set Enrichment Analysis (GSEA) of RNA-seq data comparing FGF12 cells versus controls. Bubble plot shows 14 significantly enriched pathways. Bubble size represents the number of genes in the pathway, and bubble color indicates *p*-value (FDR). (**C**) qRT-PCR validation of RNA-seq results for selected genes. Expression levels of NEAT1, MALAT1, SLC7A11, GDF15, and TP53INP1 are shown in LNCaP/Du145 FGF12 cells versus controls. Data normalized to GAPDH, presented as mean ± SD from three independent experiments. Statistical significance was determined by *t*-test; *p* < 0.05 was considered significant.

Next, we performed RNA sequencing analysis to define the transcriptome regulated by FGF12. With a fold-change threshold of 1.5 and a *p*-value cutoff of 0.05, we identified a set of 235 genes altered by FGF12 expression. The volcano plot (Figure 4A) highlights transcripts significantly modulated by FGF12, with a notable enrichment of lncRNAs, including NEAT1, MALAT1, and FTX, among the upregulated genes. GSEA was then conducted to identify coordinated pathway changes associated with FGF12 expression. Fourteen enriched pathways are shown in the bubble plot (Figure 4B). Among these, the YB1-related gene set, BASAKI_YBX1_TARGET_DN, which includes genes downregulated upon YB1 knockdown, was prominently activated with FGF12 expression. Meanwhile, the gene set, BASAKI_YBX1_TARGETS_UP, was suppressed. Given that NEAT1 and MALAT1 are members of BASAKI_YBX1_TARGET_DN, these results suggest a potential link between FGF12 and YB1-mediated post-transcriptional regulation.

In addition, pathways related to ion transport and NF-κB signaling were enriched, consistent with known roles of FGF12 in sodium channel regulation and NF-κB modulation. Moreover, pathways related to mitosis were suppressed in FGF12 cells, aligning with our previous observation of reduced proliferation rate upon FGF12 expression (Supplementary Figure S3). Finally, pathways associated with negative regulation of RNA transcription were enriched in the control group relative to the FGF12-expressing group, further supporting the selective elevation of lncRNAs in the FGF12 expression condition.

To validate the RNA-seq results, we selected five genes from the YB1-related gene set for qRT-PCR analysis. In LNCaP cells (Figure 4C, left), FGF12 increased the expression of NEAT1, MALAT1, SLC7A11, GDF15, and TP53INP1. We further examined these genes in another PCa cell line, Du145 (Figure 4C, right), where all five genes exhibited varying degrees of upregulation, consistent with the findings in LNCaP cells. These results confirm that FGF12 regulates transcriptional programs characterized by lncRNA upregulation and activation of a YB1-associated gene network, implicating that FGF12 may regulate lncRNAs to contribute to PCa progression.

### 3.5. FGF12 Interacts with YB1 in LNCaP Cells

**Figure 5:**
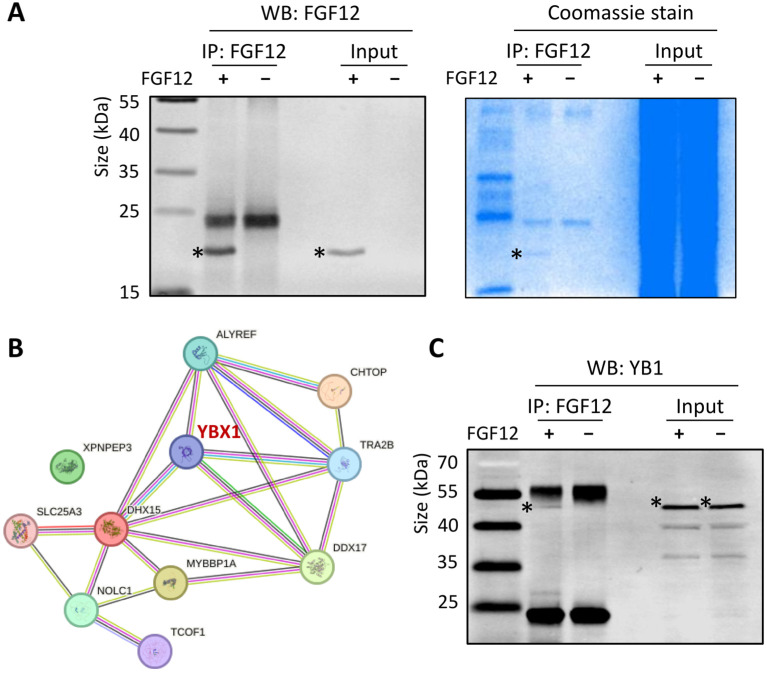
FGF12 interacts with YB1 in LNCaP cells. (**A**) Co-immunoprecipitation (Co-IP) of FLAG-tagged FGF12 in LNCaP FGF12 and control cells, detected by Western blotting and Coomassie staining. (**B**) STRING protein–protein interaction network derived from mass spectrometry analysis of proteins Co-IP with FGF12 in LNCaP cells. YBX1 appeared as a central hub in the network. (**C**) Western blotting analysis validates FGF12-YB1 interaction by Co-IP of FLAG-tagged FGF12 in LNCaP cells. All the Co-IP results were validated with IgG controls. All protein bands in the figure are marked by “*”.

To further investigate how FGF12 exerts its regulatory roles in gene transcription, we performed co-immunoprecipitation (Co-IP) using an anti-FLAG antibody in LNCaP FGF12 and control cells. Western blotting analysis confirmed successful enrichment of FLAG-tagged FGF12 in the pull-down proteins, and Coomassie blue staining revealed distinct protein bands selectively present in the FGF12 cells but absent in controls (Figure 5A). FGF12-associated proteins were further analyzed by mass spectrometry. A total of 208 candidate proteins were identified, of which 51 were exclusively detected in the FGF12 group with triplicate repeats (Supplementary Table S4). STRING network analysis revealed an enrichment of proteins involved in RNA binding, transcriptional regulation, and stress response, with YB1 emerging as a central node in the network (Figure 5B).

Given that YB1 is an RNA-binding protein with established roles in regulating tumor progression, therapy resistance, and lineage plasticity [28], we studied the protein–protein interaction between FGF12 and YB1. Co-IP followed by Western blotting with an anti-YB1 antibody confirmed a specific interaction between FGF12 and YB1 in LNCaP cells (Figure 5C). To verify the specificity of the YB1 signal, we repeated the Co-IP and Western blotting analysis by silencing YB1, which led to a marked reduction in the corresponding band, confirming that the detected signal indeed represents YB1 (Supplementary Figure S5). These findings demonstrate that FGF12 forms a protein complex with YB1 in PCa cells, supporting that FGF12 may regulate gene expression and cellular processes through YB1.

### 3.6. FGF12 Enhances YB1-Associated lncRNA Binding and Expression

**Figure 6:**
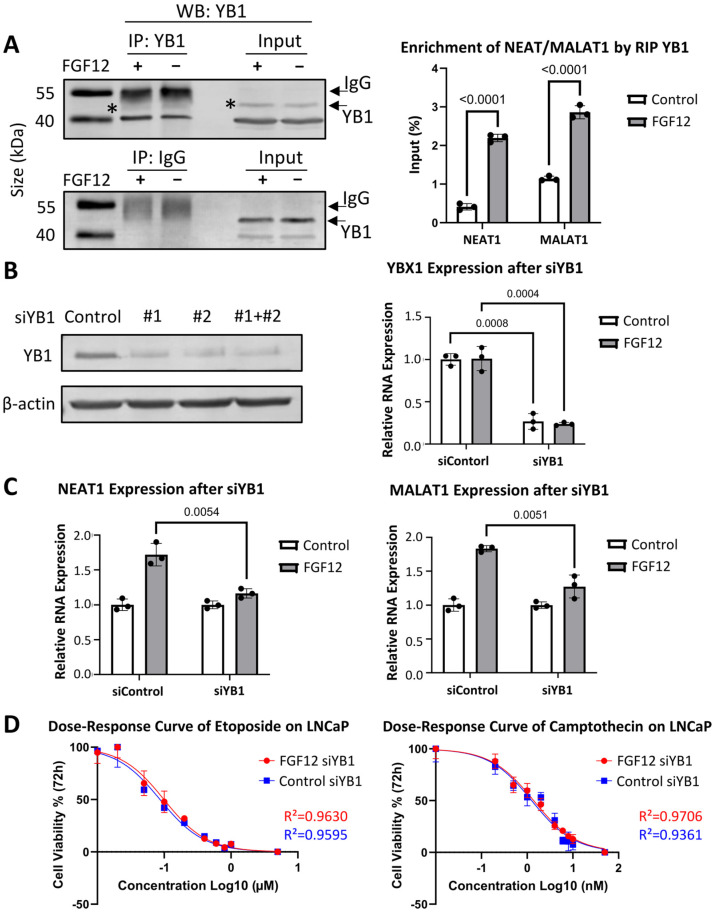
FGF12 enhances YB1-associated lncRNA binding and expression. (**A**) RNA co-immunoprecipitation (RIP) using YB1 antibody in LNCaP FGF12 and control cells. Western blotting image shows the pull-down of YB1. Enrichment of NEAT1 and MALAT1 was quantified by qRT-PCR and presented as % input relative to total RNA. FGF12 expression significantly enhances YB1 binding to NEAT1 and MALAT1. (**B**) YB1 knockdown in LNCaP cells. Western blotting analysis verifies reduction in YB1 protein following siRNA transfection (Control, #1, #2, and #1 + #2); β-actin serves as loading control (left). qRT-PCR shows decreased YBX1 expression after YB1 depletion in both control and FGF12 cells (right). (**C**) qRT-PCR shows relative fold change in NEAT1 and MALAT1 expression in control versus FGF12 cells following YB1 knockdown. The FGF12-induced upregulation of NEAT1 and MALAT1 is diminished when YB1 is silenced. (**D**) Dose–response curves of chemotherapeutic drugs (etoposide/camptothecin) in LNCaP cells (FGF12 and control) after YB1 silencing at 72 h. Data of cell viability are normalized to 0 h and 0 concentration. Non-linear regression is performed by GraphPad Prism to fit the dose–response curve. All protein bands in the figure are marked by “*”. All the data are presented as mean ± SD from three independent experiments. Statistical significance was determined by *t*-test, and *p* < 0.05 was considered significant.

Since FGF12 upregulates the expression of YB1-binding lncRNAs, NEAT1 and MALAT1, and FGF12 forms a protein complex with YB1, these results suggest that FGF12 may modulate the expression of NEAT1 and MALAT1 through YB1. We applied RNA co-immunoprecipitation (RIP) using a YB1 antibody in LNCaP control and FGF12-expressing cells. The associated RNAs were extracted to perform qRT-PCR analysis using specific primers against NEAT1 and MALAT1. As shown in Figure 6A (left), enhanced FGF12 expression did not alter YB1 protein expression, and the inclusion of an IgG control confirmed the specificity of the YB1 immunoprecipitation. CHX chase assay further confirmed that YB1 stability did not change in FGF12-expressing cells (Supplementary Figure S6). By analyzing the RIP-associated RNAs, we observed that FGF12 significantly increased the association of both lncRNAs with YB1 (Figure 6A, right). Specifically, NEAT1 binding increased from less than 0.5% input in control cells to over 2% in FGF12-expressing cells, while MALAT1 binding rose from ~1% to nearly 3%. These data confirm that FGF12 enhances the association of NEAT1 and MALAT1 with YB1.

To further confirm whether YB1 mediates the role of FGF12 in enhancing NEAT1 and MALAT1 expression, we performed RNA silencing of YB1 using two different siRNAs (siYB1#1 and siYB1#2). Western blotting confirmed efficient depletion of YB1 protein by either siRNA alone or in combination (Figure 6B, left). Knockdown with both siYB1#1 and siYB1#2 together led to a remarkable reduction in YBX1 expression in both control and FGF12-expressing cells, as shown by qRT-PCR (Figure 6B, right). Importantly, depletion of YB1 significantly attenuated the ability of FGF12 to induce NEAT1 and MALAT1 expression (Figure 6C), demonstrating that YB1 is required for the transcriptional upregulation of these lncRNAs by FGF12. To study whether YB1 mediates the PCa survival impacts of FGF12, we then depleted YB1 by siRNA in LNCaP cells expressing either control or FGF12 and challenged these cells with increasing doses of etoposide and camptothecin (Figure 6D). We found that in the absence of YB1 there is no cell survival advantage of PCa cells expressing FGF12. The IC50 values for etoposide and camptothecin became nearly identical between FGF12-expressing and control cells, 0.0992 µM vs. 0.0866 µM for etoposide and 1.388 nM vs. 1.255 nM for camptothecin. These results indicated that YB1 is an important downstream effector of FGF12 in promoting PC cell survival. Collectively, our studies demonstrated that FGF12 enhances YB1-lncRNA interactions and YB1-dependent upregulation of NEAT1 and MALAT1, thereby revealing a novel FGF12-YB1-lncRNA signaling pathway that promotes survival of PCa cells.

## 4. Discussion

In this study, we identified FGF12 as a novel regulator of cell survival for PCa cells. Our findings demonstrate an upregulation of FGF12 across different t-NEPC models and the functions of FGF12 in promoting cell survival under therapy-induced stress conditions. Mechanistically, we reported that FGF12 interacts with the RNA-binding protein YB1, enhancing YB1’s interaction with lncRNAs NEAT1 and MALAT1 and leading to their transcriptional upregulation.

t-NEPC represents a clinically significant and increasingly prevalent subtype of PCa that often emerges as a therapy-induced adaptation [3]. In this study, FGF12 was identified to be consistently upregulated in multiple t-NEPC patient biopsies, PDX models, GEMM, and in vitro cell models (Figure 1). Although IHC showed weaker protein staining than expected from RNA expression (Figure 2C), this difference may result from post-transcriptional regulation, different cohorts, and the limited sensitivity of the FGF12 antibody used in IHC analysis. Nonetheless, consistent transcriptomic upregulation across models supports FGF12 activation in t-NEPC.

Previous studies have well-characterized FGF12 as a regulator of neuronal excitability and intracellular signaling [8]. Our findings extend its biological significance to tumor survival by demonstrating its significant upregulation in t-NEPC. Of particular interest, FGF12 expression was markedly increased in LNNE cells (Figure 2), a cell line model generated through overexpression of the splicing factor SRRM4 and long-term androgen deprivation [21]. Previous studies indicate that SRRM4 promotes t-NEPC not only through genes associated with lineage plasticity and proliferation, but also through genes related to cell survival [29]. Although our data suggest that FGF12 is not sufficient to drive NE differentiation, gain-of-function of it in LNCaP cells resulted in enhanced survival under stress and reduced sensitivity to chemotherapeutic agents (Figure 3). These findings suggest that FGF12 acts as a pre-requisite survival factor for tumors to progress to t-NEPC, cooperating with NE differentiation to reinforce therapy resistance.

In this study, we also demonstrated the interaction between FGF12 and YB1 (Figure 5), an RNA-binding protein with established oncogenic functions. YB1 is a multifunctional protein associated with PCa progression and treatment resistance [28]. Under stress conditions, LIN28, a known t-NEPC promoter [30], clusters with YB1 in stress granules (SGs) to protect mRNAs from degradation [31]. YB1 has also been shown to bind lncRNAs NEAT1 and MALAT1, stabilizing them and enhancing their oncogenic functions [32,33]. Our study adds to this knowledge by showing that FGF12 strengthens the binding of YB1 to lncRNAs (Figure 6), thereby amplifying YB1-mediated post-transcriptional regulation. This represents a novel mechanism by which FGF12 exerts its oncogenic effects, not through canonical FGF receptor signaling, but by modulating the post-transcriptional regulatory capacity of an RNA-binding protein.

This study also identified NEAT1 and MALAT1 as transcriptional targets of the FGF12-YB1 axis (Figure 4). Both NEAT1 and MALAT1 are well-known oncogenic players in PCa [34]. NEAT1 is highly overexpressed in PCa and contributes to oncogenic transcription and epigenetic programming via ERα-related pathways [35]. It is also a core scaffold of paraspeckle, which is the phase-separated subnuclear condensates that regulate transcription and mediate cellular stress responses [36,37]. This suggests that FGF12 may promote paraspeckle assembly to enhance stress adaptation in t-NEPC. MALAT1, on the other hand, plays multiple roles in CRPC, including tumor aggressiveness, castration resistance, and poor clinical outcomes [38]. MALAT1 also interacts with EZH2 to enhance its oncogenic activities in CRPC [39]. By showing that FGF12 upregulates these lncRNAs, our works reveal how FGF12 may amplify oncogenic lncRNA networks central to PCa cell survival and t-NEPC development.

Beyond their roles in transcriptional control, both NEAT1 and MALAT1 have also been implicated in ferroptosis and drug resistance regulation across multiple cancer types [40,41]. Notably, our RNA-seq analysis revealed SLC7A11, a key ferroptosis suppressor [42], as one of the most upregulated YB1-associated genes in FGF12-expressing cells. This raises the intriguing possibility that FGF12 may contribute not only to cell survival under stress conditions but also to ferroptosis resistance through stabilization of NEAT1/MALAT1 and downstream activation of SLC7A11. Future studies assessing lipid peroxidation and ferroptotic sensitivity in FGF12 models will be valuable to establish this mechanistic link.

## 5. Conclusions

In summary, our studies uncover a novel FGF12-YB1-lncRNA signaling axis that plays a critical role in promoting PCa cell survival and t-NEPC progression. Through its interaction with YB1, FGF12 enhances the expression and stability of oncogenic lncRNAs, including NEAT1 and MALAT1, thereby strengthening adaptive transcriptional programs that support tumor persistence. These findings highlight the potential of targeting this axis, either at the FGF12-YB1 interface, through YB1 inhibition, or via lncRNA disruption, as a promising strategy to attenuate survival signaling in advanced PCa.

## Data Availability

The raw data supporting the conclusions of this article will be made available by the authors on request.

## Supplementary Materials

The following supporting information can be downloaded at: https://www.mdpi.com/article/10.3390/cells14221828/s1, Table S1: Primer list; Table S2: Antibody list; Table S3: Gene list of the clustering result from the five PCa cell lines; Table S4: Protein list identified from mass spectrum in FGF12-expressing LNCaP cells; Figure S1: Nine clusters of gene expression trend from the five PCa cell lines; Figure S2: Expression of NE markers and luminal markers in LNCaP control and FGF12 cells; Figure S3: Cell proliferation rate of LNCaP control and FGF12 cells. Figure S4: Western blotting analysis of ERK phosphorylation of LNCaP control and FGF12 cells. Figure S5: Western blotting analysis validates FGF12-YB1 interaction by Co-IP of FLAG-tagged FGF12 in LNCaP cells after YB1 silencing. Figure S6: Cycloheximide (CHX) chase assay determining YB1 protein stability in LNCaP control and FGF12 cells.
