# Supplementary material for: FGF12 Enhances Prostate Cancer Cell Survival via the YB1-lncRNA Axis
Source: Cells. 2025 Nov 20;14(22):1828. doi: 10.3390/cells14221828 (PMC12651396; doi:10.3390/cells14221828)

Supplementary Table S1

Primer Name	Sequence (5' - 3')
FGF12 F	ATGGAGAGCAAAGAACCCCA
FGF12 R	TCGTCCTTGGTCCCATCAAT
GAPDH F	GGACCTGACCTGCCGTCTAGAA
GAPDH R	GGTGTCGCTGTTGAAGTCAGAG
NEAT1 F	CTTCCTCCCTTTAACTTATCCATTCAC
NEAT1 R	CTCTTCCTCCACCATTACCAACAATAC
MALAT1 F	GCAGGCGTTGTGCGTAGAG
MALAT1 R	TTGCCGACCTCACGGATT
SLC7A11 F	ATGCAGTGGCAGTGACCTTT
SLC7A11 R	GGCAACAAAGATCGGAACTG
GDF15 F	CTCCAGATTCCGAGAGTTGC
GDF15 R	AGAGATACGCAGGTGCAGGT
TP53INP1 F	TTCCTCCAACCAAGAACCAGA
TP53INP1 R	AGTAGGTGACTCTTCACTGATGT
YBX1 F	GGTCCTCCACGCAATTACCA
YBX1 R	GTTGTCAGCACCCTCCATCA

Supplementary Table S2

Primary Antibody	Supplier	Cat. No.
Anti-FGF12	Proteintech	13784-1-AP
Anti-β-actin	Santa Cruz	SC-47778
Anti-Flag	SIGMA	F4042-2MG
Anti-YB1	Abcam	ab219070

Secondary Antibody	Supplier
IRDye® 680RD Donkey anti-Mouse IgG Secondary Antibody	LI-COR
IRDye® 800CW Donkey anti-Rabbit IgG Secondary Antibody	LI-COR

Supplementary Figure S1

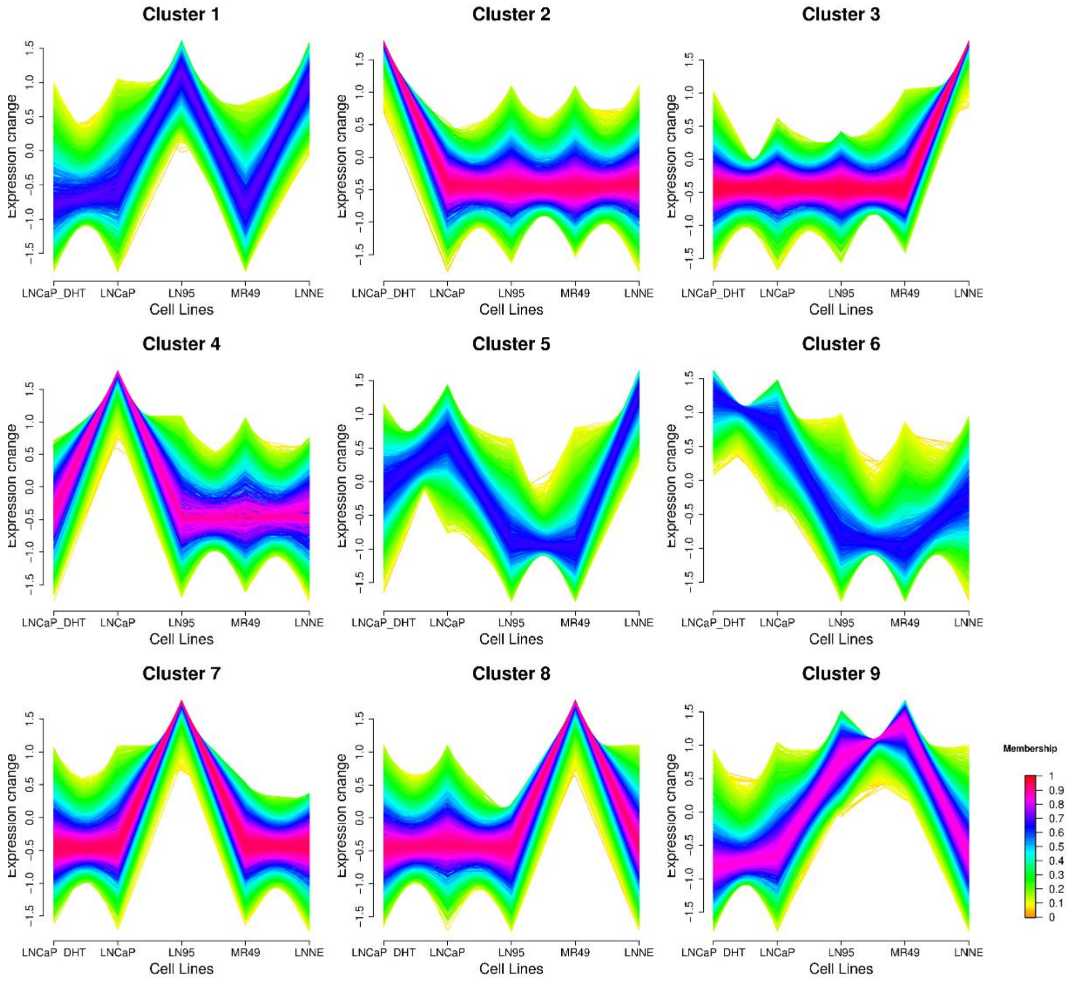

Supplementary Figure S2

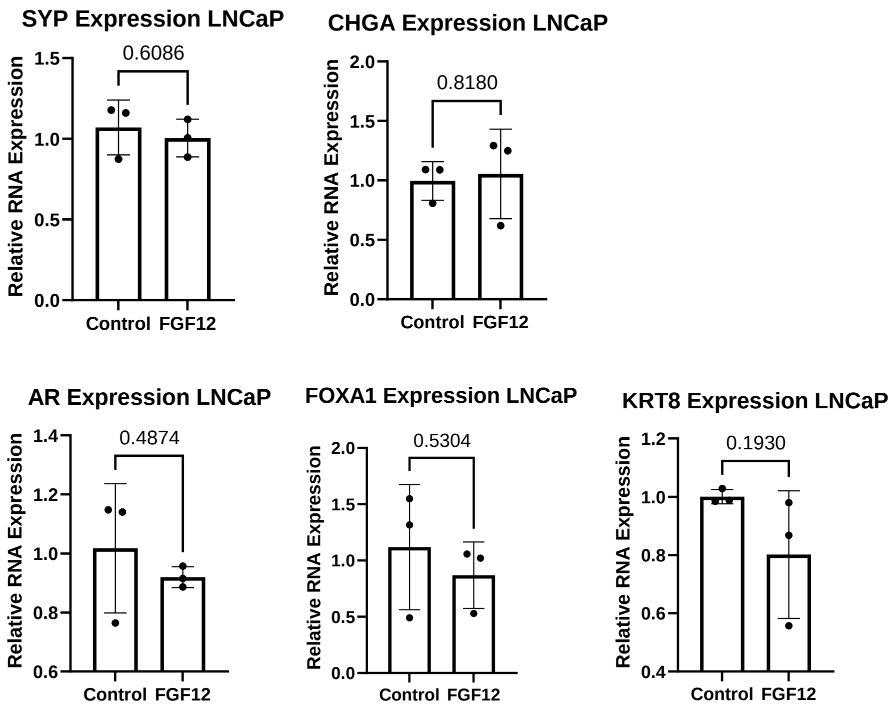

Supplementary Figure S3

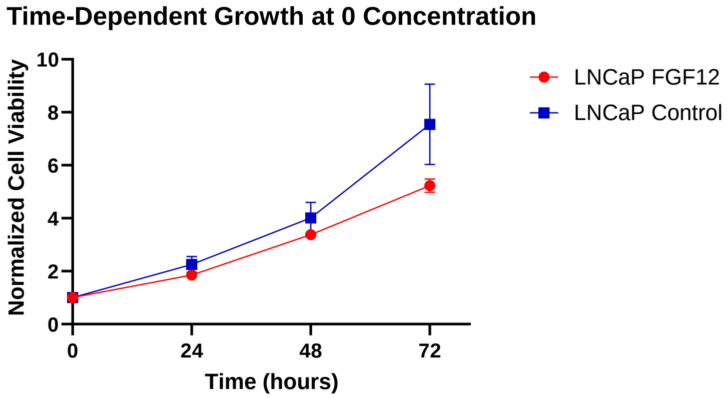

Supplementary Figure S4

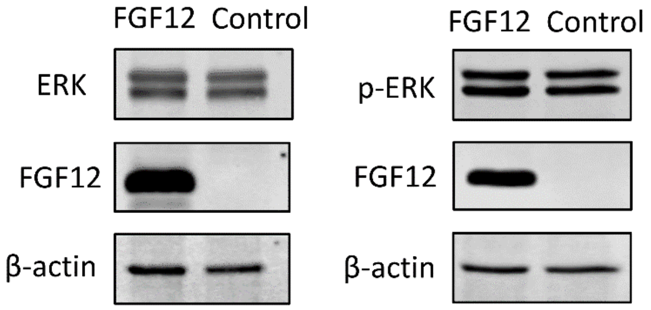

Supplementary Figure S5

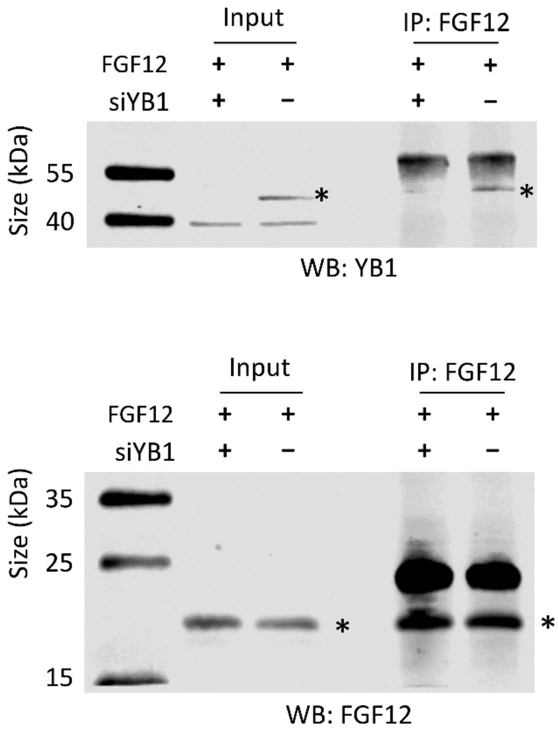

Supplementary Figure S6

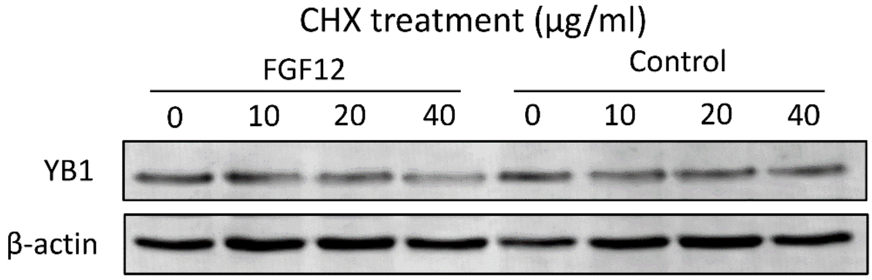

Supplement: Supplementary file 1 [file cells-14-01828-s001.zip › Supplementary Materials.pdf]
